# *In Vivo* Volume and Hemoglobin Dynamics of Human Red Blood Cells

**DOI:** 10.1371/journal.pcbi.1003839

**Published:** 2014-10-09

**Authors:** Roy Malka, Francisco Feijó Delgado, Scott R. Manalis, John M. Higgins

**Affiliations:** 1 Center for Systems Biology and Department of Pathology, Massachusetts General Hospital, Boston, Massachusetts, United States of America; 2 Department of Systems Biology, Harvard Medical School, Boston, Massachusetts, United States of America; 3 Koch Institute for Integrative Cancer Research, Massachusetts Institute of Technology, Cambridge, Massachusetts, United States of America; 4 Department of Biological Engineering, Massachusetts Institute of Technology, Cambridge, Massachusetts, United States of America; University of Michigan, United States of America

## Abstract

Human red blood cells (RBCs) lose ∼30% of their volume and ∼20% of their hemoglobin (Hb) content during their ∼100-day lifespan in the bloodstream. These observations are well-documented, but the mechanisms for these volume and hemoglobin loss events are not clear. RBCs shed hemoglobin-containing vesicles during their life in the circulation, and this process is thought to dominate the changes in the RBC physical characteristics occurring during maturation. We combine theory with single-cell measurements to investigate the impact of vesiculation on the reduction in volume, Hb mass, and membrane. We show that vesicle shedding alone is sufficient to explain membrane losses but not volume or Hb losses. We use dry mass measurements of human RBCs to validate the models and to propose that additional unknown mechanisms control volume and Hb reduction and are responsible for ∼90% of the observed reduction. RBC population characteristics are used in the clinic to monitor and diagnose a wide range of conditions including malnutrition, inflammation, and cancer. Quantitative characterization of cellular maturation processes may help in the early detection of clinical conditions where maturation patterns are altered.

## Author Summary

Red blood cell concentration (RBC), mean volume (MCV), and hemoglobin content (MCH) are routinely measured in the complete blood count, a fundamental clinical test essential for the screening, diagnosis, and management of most diseases. Variation in MCV and MCH is associated with many important clinical conditions, but we do not understand the mechanisms controlling these red blood cell physical characteristics. Vesicle shedding is thought to be most important, but we show here that a dominant role for vesicle shedding violates empirical geometric and biophysical constraints. An additional unknown process must be primarily responsible. We show that this important unknown process must be coupled to changes in RBC surface area, and we quantify the magnitude of its effects.

## Introduction

A typical red blood cell loses 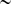
30% of its volume and 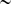
20% of its intracellular hemoglobin (Hb) over the course of its 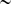
100-day lifespan in the circulation [1]–[6]. These physical characteristics control the RBC's ability to fulfill its main function of delivering oxygen to the tissues. With steady state production and recycling rates of about 2.5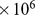 cells per second in an adult human, these processes must be tightly regulated, but despite RBC availability and many years of research [7] the mechanisms for these volume and hemoglobin loss events are not clear [8].

The complete blood count includes RBC population characteristics and is used to diagnose and monitor almost all diseases and medical conditions. These RBC characteristics are determined in large part by maturation events. Quantifying RBC maturation would help diagnose and monitor many pathologic conditions [9], [10]. Nutritional deficiencies, inflammation, and cancer often lead to changes in the circulating population of RBCs.

**Figure 1:**
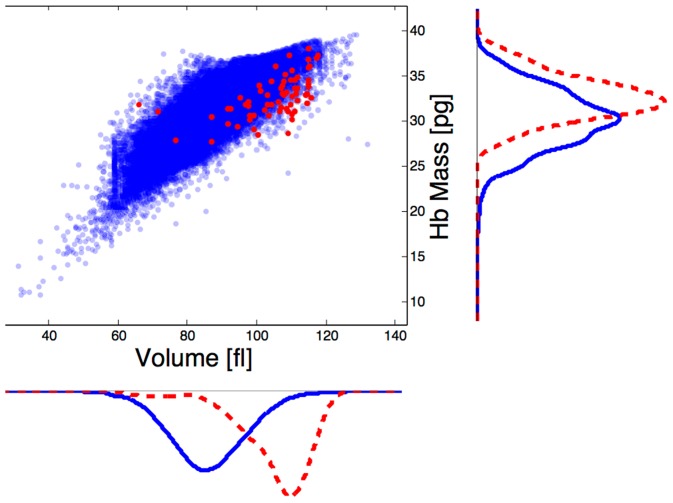
Single-cell measurements of cell volume and hemoglobin (Hb) mass content, both for the general RBC population (blue) and for some of the youngest cells (reticulocytes, in red). Both volume and Hb are higher in the young cells. Measurements were performed on 38,545 cells from a healthy individual using a Siemens Advia 2120 automated clinical hematology analyzer [30].

RBCs shed vesicles as they circulate, and this process is thought to dominate the changes in the RBC physical characteristics occurring during maturation (see e.g., [11]–[14]). Because vesicle formation can not be monitored directly in vivo, theoretical studies are essential. To investigate the role of vesiculation in RBC maturation we take advantage of powerful new single-cell measurements (see e.g., [15]–[18]). Despite the large number of techniques measuring RBC single-cell characteristics, there are not many theoretical studies integrating these data. We develop a model combining biophysical considerations with measurements of volume and hemoglobin content (as in Figure 1). The model predicts RBC dry mass and dry density characteristics (i.e., mass and density of non-water cellular content), and we use measurements of those quantities to validate the models and to define the physical requirements for RBC maturation processes.

Figure 1 shows the variation in volume and Hb mass for some of the youngest cells (<2 days old) called reticulocytes, identified by RNA staining [19], and the total RBC population from one healthy human. In Table 1 we summarize our estimates for volume and Hb loss in the transition from reticulocyte to mature cell.

**Table 1:** The changes in volume, Hb mass, and concentration in healthy adult humans (*n* = 21).

	mean change^1^	95% CI	% change
Volume lost	16.4 (fl)	(15.5, 17.4)	−15.6%
Hb mass lost	2.9 (pg)	(2.6, 3.3)	−8.8%
[Hb] increase	2.4 (g/dl)	(2.1, 2.8)	7.7%

1 Formally, 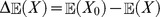, where *X*, (*X*_0_) is respectively, (initial) volume, Hb mass, or concentration (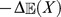, for [Hb]).

The change is the difference between means of reticulocytes (red dots in Figure 1) and the total population (blue dots in Figure 1).

We first propose a theoretical framework to investigate the effects of vesiculation on biophysical properties of RBCs as they age. In order to apply the theory we estimate the vesiculation rate from existing empirical data. We then describe the requirements for the volume reduction process, for the hemoglobin reduction process, and for the surface area reduction process. We show that vesiculation cannot account for all the volume and Hb lost by the RBCs, as the sum of volume lost or Hb mass lost in the vesicles is considerably smaller than what is lost by the cells. We find that vesiculation can explain the surface area loss and that surface area loss must be coupled to the hemoglobin loss in order to explain the observed dry density profile of red blood cells.

## Results

### Theoretical analysis of the biophysical-effect of vesiculation on a single RBC

Here we propose a family of stochastic processes that describes how the biophysical properties of a cell change as it ages and sheds vesicles in a leak-less process where all mass lost from the cell is assumed to be in the vesicles.

The model describes how a property 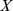 e.g., volume, mass, etc., changes when the quantity 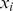 is lost in a single vesicle. Thus, 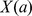 (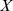 at age 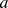) is the difference between the initial value 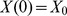 and the total quantity lost in vesicles,
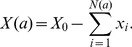
(1)



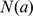
 is a Poisson process with constant rate 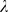, counting the number of vesicles lost by age 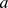. We assume that 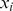 is independent of age and of 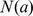, as indicated by analysis of vesicles from stored blood [20]. Thus, for simplicity our first approximation of the reduction term is a compound Poisson stochastic process [21]. Using these assumptions we can calculate the expectation with respect to 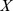 at age 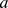:
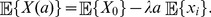
(2)


Since the population of RBCs has mixed ages, we treat the age 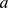 as a random variable, and treat 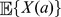 as the conditional expectation of 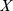 given that the cell age is 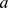: 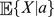. Taking a second expectation, now with respect to age, with 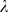 and 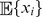 assumed to be independent of 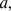 we get:
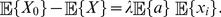
(3)


This analysis allows inference even from crude measurements, as it only requires the mean of the initial population and that of the general population, to calculate the difference. A more detailed analysis and simulations (using Poisson sample paths, see e.g., [22]) are discussed in the materials and methods. In order to apply the model we need to estimate the characteristics of vesiculation.

### Estimation of *in vivo* vesiculation rate from steady state vesicle measurements

Our knowledge of the vesiculation process is summarized by two parameters: the vesicle size distribution (vesicle size is quantified here either by volume, 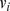, or by radius, 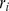 since we assume it is a sphere), which has been measured by atomic force microscopy (AFM) [23]–[25] and recently also by a micro-nuclear magnetic resonance (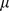 NMR) system [20], and the vesiculation rate, 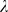. Direct estimation of vesiculation rate is not feasible in physiological conditions because currently a specific RBC cannot be monitored repeatedly in vivo. Here we approach the rate estimation by modeling dynamics and analyzing steady states. The vesicle count in a blood sample reflects the balance between production and clearance, formulated as
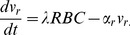
(4)



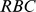
 is the red blood cell concentration (cells/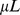) and 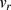 is the vesicle concentration (vesicles/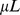). The model parameters are the average vesiculation rate 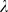 (vesicles/cells/day) and the clearance rate 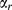 (1/day). If we assume steady state (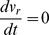), then
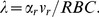
(5)



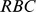
 is measured routinely in the clinic, and there are values of 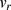 in the literature. The clearance rate 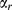 can be estimated by combining vesicle labeling studies [26] and Eq. (4) (see Table 2 and materials and methods). In Table 2 we collect estimates for 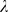 obtained from measurements of vesicle and RBC counts and Eq. (5). We find that estimated 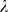 can span two orders of magnitude (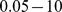 per day) in healthy humans.

**Table 2:** Values for the vesiculation rate (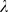).

vesicles/cell/day 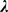	vesicles/*µL v_r_*	ref
8.97	30, 199±19, 686^1^	[31]
0.62	2100^1^	[32]
0.05	169^1^	[11]

1 The RBC value used is 4.8±0.58×10^6^ cells/*µL* from [31].

The values of 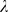 are obtained by using Eq. (5) and the estimated vesicle clearance rate (*α_r_* = 1425 per day) (see materials and methods for details on *α_r_*).

### Volume reduction due to vesiculation

In this section, we use the estimated average vesiculation rates (in Table 2) and empirical vesicle sizes (see materials and methods) to show that most (>80%) of the volume reduction during cell aging cannot be explained by vesiculation. We model the cell volume reduction using Eq. (1), replacing 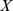 with 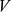:
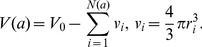
(6)



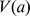
 is the volume of a cell at age 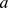, and 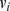 is the volume of the *i*th vesicle (approximated as a ball with radius 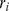), leading, as in Eq. (3), to the following relationship between 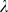 and vesicle size:
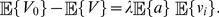
(7)


**Figure 2:**
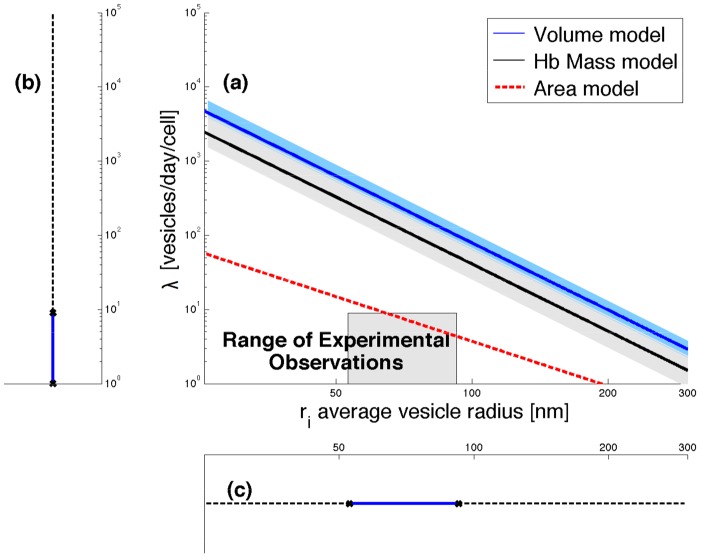
Measurements versus predicted change of volume, Hb mass, and surface area due to vesiculation. (a) Human blood measurements (n = 21) are used to investigate the relationship between vesicle radius (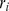) and shedding rate (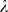) as predicted by the volume loss model (log of Eq. (7), blue line (solid line for mean, shaded area for range) and Hb mass model (log of Eq. (8), black line) versus the observed parameter values (gray area). Average RBC age assumed to be 50 days. The area model (log of Eq. (9), dashed-red) predictions are obtained using data from Table 4. (b) the range of average vesiculation rate (Table 2) and (c) average vesicle size (see materials and methods for details.)

We estimate 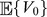 from the young reticulocyte population (red dots in Figure 1), assumed to be at age 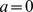, (using the Advia reticulocyte dye intensity, we take cells associated with the highest third). We estimate 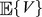 from the total RBC population. The maximal estimate for 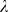 is 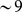 (vesicles/cell/day) and would require an average vesicle radius >185 nm to account for the total volume lost (as reported in Table 3). That vesicle radius is twice as large as any reported average vesicle measurement (see materials and methods). The reported average vesicle radii are 53–93 nm, and would require 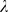
>75, which is 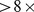 larger than the largest empirical estimate of 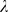. In Figure 2 we can see the large difference between the model predictions (log of Eq. (7) in blue) and the range of experimental observations based on blood cell measurements from 21 healthy human adults (solid lines mark the means, and the accompanying shaded regions mark the ranges). The large difference between the range of observed 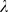 and 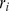 values and the values predicted by the model indicates that vesiculation can explain only a small fraction (<20%) of the observed volume loss (see Table 3). In the materials and methods we conclude the same using independent data and a geometric argument.

**Table 3:** Estimated fraction of volume and Hb lost (mean ± std) by vesiculation as a fraction of total volume or Hb lost during maturation.

	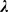 1 = 4	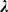 = 10
Volume lost in vesicles %	5.2 (±0.7)	13.0 (±1.7)
Hb lost in vesicles^2^ %	10.5 (±2.7)	26.1 (±6.6)

1 For these values (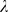 = 4, *r_i_* = 100) we get ∼100% for the fraction of surface area lost via vesiculation.

2 Vesicle [Hb] is that of the shedding cell, which is later referred to as model 1.

We assume a vesicle radius of 100 nm (the 90th percentile).

### Hemoglobin mass reduction due to vesiculation

We now utilize Eq. (1) to describe the Hb mass reduction. The Hb mass in a cell of age 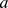 (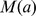) is the difference between the initial Hb mass (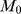) and the total mass lost in vesicles (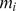 is the Hb mass in a single vesicle). The relation between mass lost and vesiculation parameters, as in Eq. (3), is given by
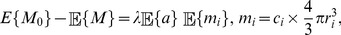
(8)where 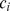 is the Hb concentration, [Hb] in the vesicle. Combining blood measurements of cellular Hb mass (as in Figure 1) with Eq.(8) we obtain the predicted relation between 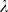 and vesicle size in Figure 2 (black line for the mean prediction). The vesiculation model can explain only 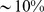 of the total Hb lost, and at most 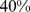 under extreme assumptions (see Table 3). Thus we conclude now that processes other than vesiculation must be involved in RBC maturation.

In our model the Hb mass in the vesicles is proportional to the mean Hb concentration, 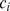, in the shedding cells (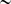
30–35 g/dL). If the vesicle [Hb] were at least five times larger, on average, the vesiculation could explain the total Hb loss, but such Hb concentration is not physically possible and would also require an unknown process to pack the Hb into the vesicles. Alternatively, measurements may underestimate the small vesicle fraction, but we show later that an increased small vesicle fraction is inconsistent with dry mass measurements, suggesting that an additional mechanism is indeed involved in the Hb reduction.

### Surface area reduction due to vesiculation

The evolution of the surface area, and the relation between area lost and vesiculation parameters, as in Eq. (3), is given by
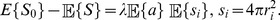
(9)



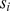
 is the membrane surface area of a sphere-shaped vesicle. We use the values from Table 4 to estimate the area loss and Eq. (9) for the predictions in Figure 2 (dash-red line). We find that in the case of surface area loss, vesiculation alone is a sufficient mechanism for the surface area loss, given the constraints on vesicle sizes (average radii are 53–93 nm) and 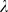 (4–13 vesicles/day).

**Table 4:** The changes in cell membrane area, volume, and Hb mass for 2 human subjects.

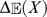 1	mean	% change ( 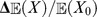 )
Subject 1		
Area lost	23.3 (*µm* ^2^)	−13.7%
Volume lost	16.5 (fl)	−14.3%
Hb mass lost	3.8 (pg)	−9.9%
Subject 2		
Area lost	20.4 (*µm* ^2^)	−11.9%
Volume lost	15.4 (fl)	−13.1%
Hb mass lost	2.7 (pg)	−7.3%

1 This data is adapted from Table I of [3], calculated as the difference between averages of reticulocytes and all RBC parameters, (in the terminology of [3] the difference between ‘total reticulocytes’ and ‘whole blood’.)

### Dry mass reduction due to vesiculation

**Figure 3:**
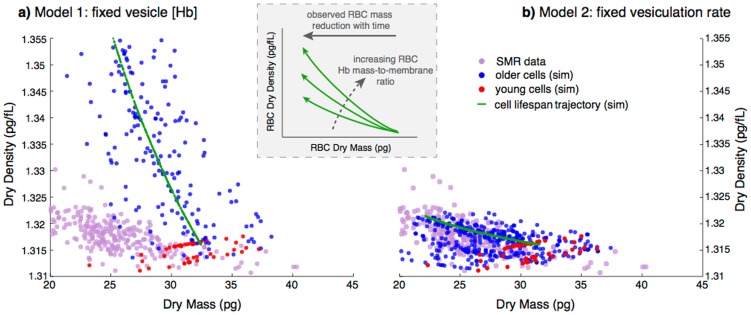
Initial values of cellular dry mass and density (red dots) are mean-matched to the upper 20% of dry mass values as measured by the SMR (purple dots). Cellular dry mass and density evolve according to models 1 and 2 described in the main text. The green line is a detailed path of an example cell, the other paths are randomly sampled (blue dots) to match the sample size of the measured SMR data. (**a**) The vesicle [Hb] is identical to that of the shedding cell, and this model then requires 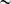
10 times more vesicles per cell than empirical estimates. (**b**) The Hb mass lost is divided between a fixed number of vesicles (N = 550) for each cell and this model then requires an infeasible vesicle [Hb] of 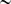
260 g/dL. (**Inset**) Dry density is determined by the Hb mass-to-membrane ratio. The diagram shows the expected change in a cell's trajectory when the ratio increases, which will happen when the vesiculation rate increases while the total Hb mass loss remains constant, leaving the mature cell with less membrane.

We can decouple the hemoglobin mass dynamics from the volume dynamics by measuring cellular dry mass (non-water mass) and cellular dry density (density of non-water constituents). We now integrate models of Hb reduction and surface area reduction (Eq. (8) and Eq. (9)) resulting in Eq. (15) to study the changes in the cell's dry mass and density during maturation. A cell's Hb mass is estimated to be about 95–97% of its dry mass [27]. We use newly available measurements of single-cell dry mass and dry density (referred to as SMR) [16]. Figure 3 shows cellular dry mass and density (purple dots) for an RBC population obtained via the SMR. Dry density shows very little variation with dry mass, despite the large change in dry mass over the cell's life, (dry mass has a coefficient of variation of 16.9% versus a 0.3% for the dry density).

We assume the following: I. RBC dry mass consists of membrane and Hb. II. The Hb and membrane density 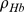 and 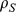 are constant during the cell life, and 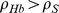 (see Table 5). III. The membrane mass and volume are linearly related to the membrane surface area, see Eq. (14). Rewriting Eq.(8), using the number of vesicles 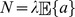:
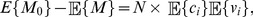
(10)shows that the lost Hb mass is determined by the number of vesicles, their Hb concentration, and their volume. Details on the simulations are given in the materials and methods. Briefly, we simulate the evolution of the cell's dry mass-density, using Eq. (15), Advia data (see Figure 1), and a random sample of vesicle sizes (see Materials and Methods) under two sets of assumptions.

**Table 5:** Parameter values.

Parameter	value	ref
Surface membrane density^1^, *ρ_s_*	1.15 (pg/fl)	[33]
Membrane volume/area, *v_s_*	1.009 (fl/*µ* m^2^)	[33]
Hb density, *ρ_hb_*	1.36 (pg/fl)	[34]

**Model 1**: Fix the vesicle's Hb mass by setting the vesicle [Hb] equal to that of the shedding cell.

**Model 2**: Fix the number of vesicles at the highest level consistent with empirical evidence (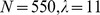).

We compare the simulation results to the SMR dry mass-density measurements. Figure 3a shows that when young cells (red dots) evolve according to model 1 their dry density increases (see blue dots), inconsistent with the experimental observation (purple dots), and determines an N (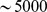) that is inconsistent with empirical estimates (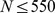). We therefore exclude the possibility that we are underestimating the number of small vesicles. When young cells evolve according to model 2 (Figure 3b) their dry density trend fits the experimental data, demonstrating that the rate of Hb loss matches the rate of membrane loss during aging. However, this model requires the vesicle Hb content to be physically unrealistic, based on the values in Table 5 and [26]. The consistency of the model with the data implies that some of the Hb is lost via a mechanism other than vesiculation, and that mechanism is synchronized with vesiculation. One mechanism that can generate such synchronization between the surface loss and hemoglobin loss is a leaky vesiculation, in which some hemoglobin is lost to the surroundings during the process of vesicle release.

## Discussion

We establish that while vesiculation alone can explain the observed membrane lost during RBC maturation, it cannot explain all the Hb or volume lost. There must be an additional process to explain the remaining 60–90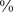 of the volume and Hb reduction occurring during RBC maturation.

Dry density is determined by the ratio of Hb mass and cell membrane surface area. Because we see very small changes in cell dry density despite large changes in dry mass, we conclude that changes in Hb mass must be coupled to changes in cell membrane surface area. We suggest that the unknown process(es) responsible for up to 90% of the Hb mass and volume reduction are therefore physically linked to the vesiculation-based changes in surface area. It is possible that the unknown processes involve leaky vesiculation, intracellular degradation and excretion and/or interactions with white blood cells or other cells. RBC indices are used in the clinic to monitor and diagnose a wide range of conditions. Our work helps focus future investigation of molecular mechanisms of RBC maturation whose characterization may help in the early detection of clinical conditions where the maturation pattern is altered.

## Materials and Methods

### Estimating the vesicle clearance rate

**Figure 4:**
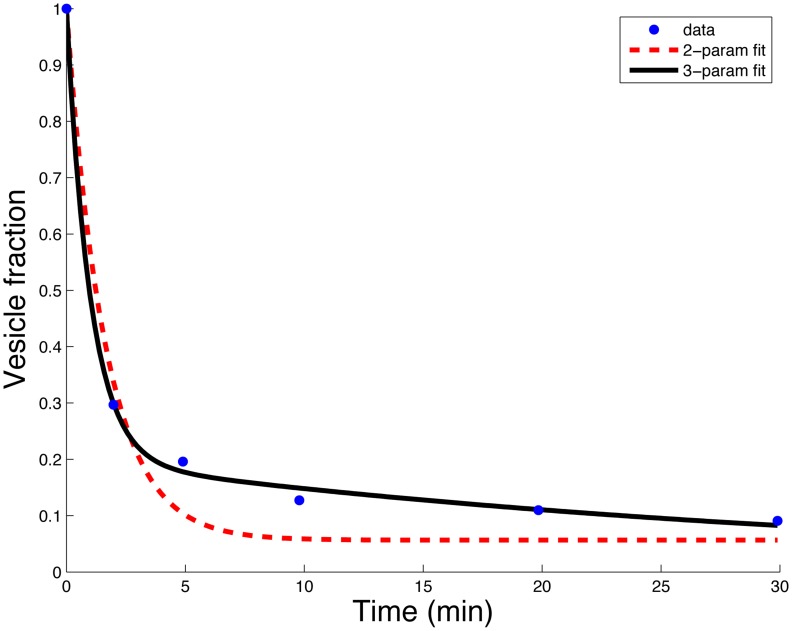
Vesicle clearance pattern in rats (blue dots). For the 2-parameter model in Eq. (11) (red/dashed-line) the estimates are 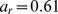 and 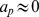 per minute (with 95% confidence interval (CI) of 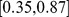 for 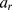, and for 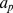 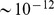). For the 3-parameter model in Eq. (12) (solid/black curve) the estimates are 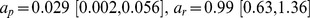 per minute, and 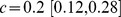.

We estimate the vesicle clearance rate from data reported in [26], where vesicle labeling studies were performed in rats. This data is equivalent to a trajectory of the system in Eq. (4) with 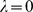, allowing us to get a physiological estimate of 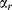. In those experiments the RBC vesicles were reported to be highly enriched relative to platelet vesicles (16.7∶1). Thus, the vesicle fraction data appearing in Figure 4 (blue dots) is the sum of RBC vesicles (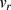) with some small initial fraction of platelet vesicles (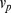). The results of the experiments describe the total vesicle concentration over time: 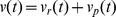. We assume here a linear ODE model for the clearance of vesicles, without interaction, namely, taking Eq.(4) for 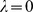 twice, replacing 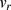 and 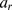 with 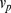 and 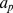 to form the clearance model for platelets. The sum of the solutions of these two models gives the total vesicle concentration over time,
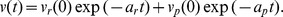



The data reported in [26] is the remaining fraction of vesicles in the circulation and not absolute counts. Thus, we derive the clearance parameter from the remaining fraction 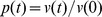, where 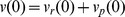. The reported purity of RBC derived vesicles versus total vesicles is 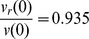 (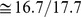) and similarly for the platelet derived vesicles 
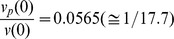
. Thus,
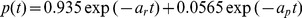
(11)and we have two parameters to estimate, 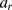 and 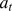. The result of the fitting of those two parameters appears in Figure 4 (red/dashed curve). Ignoring the reported purity, we fit 3 parameters using,
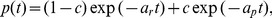
(12)and improve the fit, as expected, (see black/solid curve in Figure 4). The fitted value of 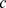 raises the hypothesis that the reported protocol leads to 80% enrichment of RBC vesicles.

Modeling note: in a linear model, different initial loads behave in the same way. The current data does not allow us to probe this question. Future vesicle labeling studies should include different doses of vesicles to test whether the response is sensitive to the initial load or not.

### Upper bound on vesiculation rate from geometric constraints

Here we investigate the second vesicle characteristic, the vesiculation rate, based on the assumption that a sphere-shaped red cell will be cleared as it cannot deform. Given an initial cell with a maximum volume of 110 fl (Table 6) which has surface to volume ratio of 1.7 [3] the initial surface area will be 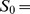
187 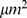. Assuming that cells cannot get smaller than 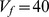 fl and that those cells are spheres leads to an estimate of the total area lost during the cell life in circulation to be 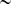 130 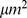. If this area is distributed to a random sample of vesicles (drawn from an empirical distribution [20] with averaged radius (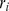) of 83.5 nm) over the course of 120 days (Age), the cell is estimated to lose 12 vesicles per day:
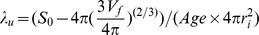



**Table 6:** The raw data used to calculate the values in Table 1.

	Volume (fl)		Hb mass (pg)		[Hb] (g/dl)	
Subject	Reticulocytes	total	Reticulocytes	total	Reticulocytes	total
1	103.6	86.3	34.2	30.3	33.5	35.2
2	109.8	88.6	33.3	30.2	30.6	34.2
3	104.7	91.2	33.8	31.7	32.7	34.9
4	103.6	85.5	34.2	30.4	33.2	35.8
5	111.1	95.0	36.8	32.2	33.4	34.1
6	107.8	91.6	33.2	30.7	30.9	33.6
7	114.8	96.8	35.1	33.0	30.7	34.3
8	105.3	89.2	33.3	31.0	31.8	35.0
9	97.3	84.4	29.3	26.8	30.3	31.9
10	101.7	86.6	33.6	30.9	33.2	35.9
11	100.5	82.8	32.3	28.5	32.7	34.6
12	107.6	93.1	34.9	32.6	32.7	35.2
13	102.5	84.4	33.1	29.3	32.5	34.9
14	105.2	88.1	32.2	29.8	30.8	33.9
15	104.1	90.2	32.0	29.7	31.1	33.0
16	108.1	91.0	33.4	29.7	31.0	32.8
17	107.0	90.3	33.4	30.8	31.3	34.3
18	111.2	95.0	34.0	31.0	30.8	32.7
19	103.3	83.8	34.0	30.2	33.3	36.2
20	103.6	90.9	32.4	30.7	31.5	33.8
21	103.0	86.1	32.8	30.2	32.0	35.2

**Figure 5:**
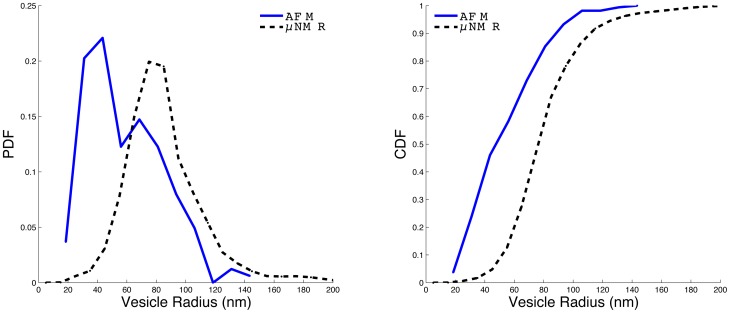
Vesicle size distribution. The AFM data is from [25] while the 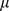 NMR data is from [20]. (left) probability distribution functions (PDF) and (right) cumulative distribution functions (CDF). The AFM histograms had a bin size of 12.5 nm and the counts of nano-vesicles and micro-vesicles were merged. The 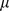 NMR had a bin size of 10 nm.

The above values, in particular 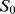 and the use of radii sampled only from the PDF of 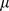 NMR (larger vesicles), yield a conservative vesiculation rate estimate. If we choose 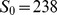 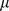
m^2^, 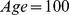, and vesicles radii sampled from the PDFs measured with both AFM and 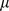 NMR (see Figure 5), sampled with equal weight, we obtain an upper bound on the vesiculation rate of 27 vesicles per day. The average rates are lower. This analysis thus support the vesiculation rate estimates in the main text using independent data and reasoning.

### Geometric constraints on volume reduction

We argue here that it is geometrically impossible to explain the volume lost only by vesiculation, supporting the conclusions obtained in the main text using our stochastic processes theory, with independent data. Previous measurements [3] comparing young RBCs to the total population found that the percent change in area is similar to the percent change in volume. Formulating mathematically,
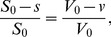
(13)where 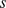 and 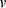 are the total surface and total volume lost. Using empirical values from Table 4 the equality in (13) holds with less than 1% error. If vesiculation is responsible for all volume and surface area loss, then 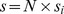 and 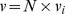, where N is the total number of vesicles (assumed to be of fixed size). The above relationship requires that the surface to volume ratio remain constant for the entire RBC lifetime (as in Table I of [3]). However, this relationship implies that the surface to volume ratio of the cell (in initial state) is identical to that of the vesicle (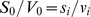). Given a cell with volume V and surface area S, we define 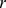 as the solution of 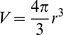. From the isoperimetric inequality in 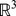 [28], we know that given a volume 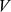, the minimal surface area 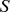 bounding 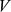 is a sphere . Hence, in general, for a given volume, 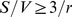 (with equality for a sphere). For a vesicle, it is reasonable to assume a spherical shape so 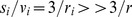 since 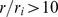. For example, if 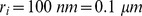, then 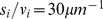. Estimates of 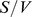 for RBCs are around 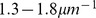, more than an order of magnitude smaller, and thus inconsistent with vesiculation as the only volume loss mechanism.

### Estimating vesicle size

Here we report the findings of others regarding vesicle sizes, which together with the vesiculation rate complete the current characterization of the vesiculation process required in order to apply the theory in the main text. We reproduce the vesicle size distribution from [25] figure 1D (referred to as AFM) and the vesicle distribution from blood stored in blood bank conditions [20] (referred to as 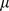 NMR). Notice that here size is radius (to be consistent with the mathematical analysis) rather than diameter as is used more commonly in the literature, and specifically in [20], [25]. The vesicles in [25] were induced using Ca^++^/ionophore A23187 (Sigma, St Louis, MO). Smaller vesicles are referred to as ‘nano-vesicles’ and the larger ones are referred to as ‘micro-vesicles’. We use their counts and merge the two types to form a vesicle size probability density function, pdf, (Figure 5, left, blue line) and cumulative distribution function, cdf, (Figure 5, right, blue line). The AFM cdf shows that the 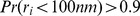 (for the 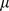 NMR, it is 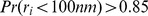) which is the mean vesicle size we used in some of the simulations (it is above the 95% CI of the mean even for the 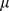 NMR data). Even if the vesicle size is dependent on the cell age, we are using a realistic sample size or a larger-than-average vesicle size, and thus we expect that a simple age dependence would not allow explanation of the results based on vesiculation alone. Note that a small number of very large vesicles during the RBC life with large volume (e.g., total of 15 fl) might explain the volume loss but would be inconsistent with measured steady state vesicle concentrations.

### Model and simulation of dry mass evolution

The cell dry density calculations require the use of the membrane volume 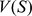 and mass 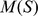, which are assumed to follow:
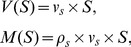
(14)



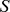
 is the membrane area. This relation is assumed to hold independently of cell age, using the values of 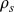 and 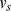 from Table 5.

The following conditions are used in both model 1 and 2. We estimate the Hb mass lost from measurements as 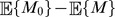, that is, the difference between the mean Hb mass of the young RBCs and that of the total RBC population (Advia data as in Figure 1). Then we generate a Poisson sample path [22] and use a random sample of vesicle radii from the distribution in Figure 5. With the assumption that the vesicles are spherical, we can infer the vesicle surface area and volume. The simulations require initial values of Hb mass and surface area. Hb mass is obtained per sample from the Advia clinical analyzer, and the initial surface area is volume-matched to the data in [29]. By ‘volume-matched’ we mean that for each young cell with known Hb mass and volume, we find the cell with the closest volume in the volume and surface area data from [29], and use that surface area to form the Hb mass-surface area pair. We use Eq. (8) and Eq. (9) for the evolution of the Hb mass and surface area, their sum for the dry mass, and Eq.(15) for the dry density.

The trend in the dry mass and dry density is predicted by combining the data from the Advia clinical blood analyzer and volume-matched surface area from [29]. The SMR data is a third data source, and each data set may have a fixed offset depending on the particular device calibration (see Table 7 for summary on data sets used). The data in Figure 3 is obtained by matching the means of the dry mass and dry density of the initial data used in the simulation to that of the top 20% of the measured SMR dry mass (offset of 14.6% for the mass and 0.9% for the density). The offset does not affect the relation between the dry mass and dry density, as we are just adding a constant.

**Table 7:** Summary of major data sources used.

Parameter	Measurement	Source/Method	Comment
Cellular volume and Hb mass	RBC single cell	Advia - light scattering [30]	reticuloctyes/all RBCs separately
Cellular surface area	RBC single cell	HEMA - microfluidic device [3]	reticuloctyes/all RBCs separately
		microscopy of microfluidics [29]	
Cellular dry mass and dry density	RBC single cell	SMR - inertial [16]	
Vesicle sizes	Single vesicle	AFM [25]	measuring the vesicle diameter
		*µ* NMR [20]	
vesiculation rate	Count labeled vesicles	Capture, label, reinfuse, and sample experiment [11], [26], [31], [32]	measure the fraction of labeled vesicles repeatedly over time
Average RBC age	Count labeled RBC	Capture, label, reinfuse, and sample experiment [26]	measure the fraction of labeled RBC repeatedly over time

See Materials and Methods for description of the techniques.

Note that the assumption of a ball-shaped vesicle is conservative, as it leads to a lower bound estimate on the actual increase in density. Any other shape will require more vesicle surface area to contain the same amount of volume, requiring the shedding cell's density to increase more than in the case of a ball-shaped vesicle.

### Analysis of dry density along a cell's trajectory

The following calculations show under assumptions in Eq. (8) and Eq. (9), and in particular 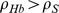, that the shedding of each vesicle by a cell requires that the cell's dry density must increase. Using Eq. (10) we calculate the cell dry density:
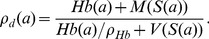
(15)


An increasing trend in density along a trajectory requires that if 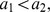 then 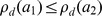 (with strict inequality for at least some of the ages). We now look at the effect of shedding a single vesicle. This analysis is sufficient due to the independent increment property of the proposed model. If 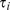 is the time of shedding of the 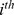 vesicle, we compare 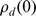 to 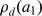 for 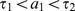 (i.e., 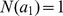). Rearranging Eq. (15) we get 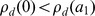 if
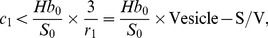
(16)where 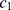 is the [Hb] in the vesicle. Eq. (16) gives an upper bound on the vesicle Hb concentration. The bound is calculated from the initial cellular Hb mass and membrane surface area, as well as the vesicle's radius. Taking 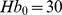 (pg), 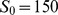 (*µ*m^2^) and 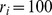 (nm), the bound on the concentration is 600 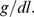 For reference, [Hb] in human RBCs is never outside the range 20–50 g/dl, and it is physically impossible to achieve such high [Hb] (based on the values in Table 5). The immediate conclusion is that we always have increasing dry density along a cell trajectory. This result could be demonstrated experimentally if it were feasible to monitor a single RBC in circulation for at least several hours.

We thus find that cell dry density must increase monotonically with each vesicle shed. This analysis of dry density along a path shows that the difference in the two models (as seen in Figure 3 a-b) is quantitative and not qualitative, since both models have an underlying increasing trend. The second model (see Figure 3b) has a milder increase per vesicle and fewer vesicles along the trajectory, which make the trend less apparent against the background population variation.

### SMR data and modeling

**Figure 6:**
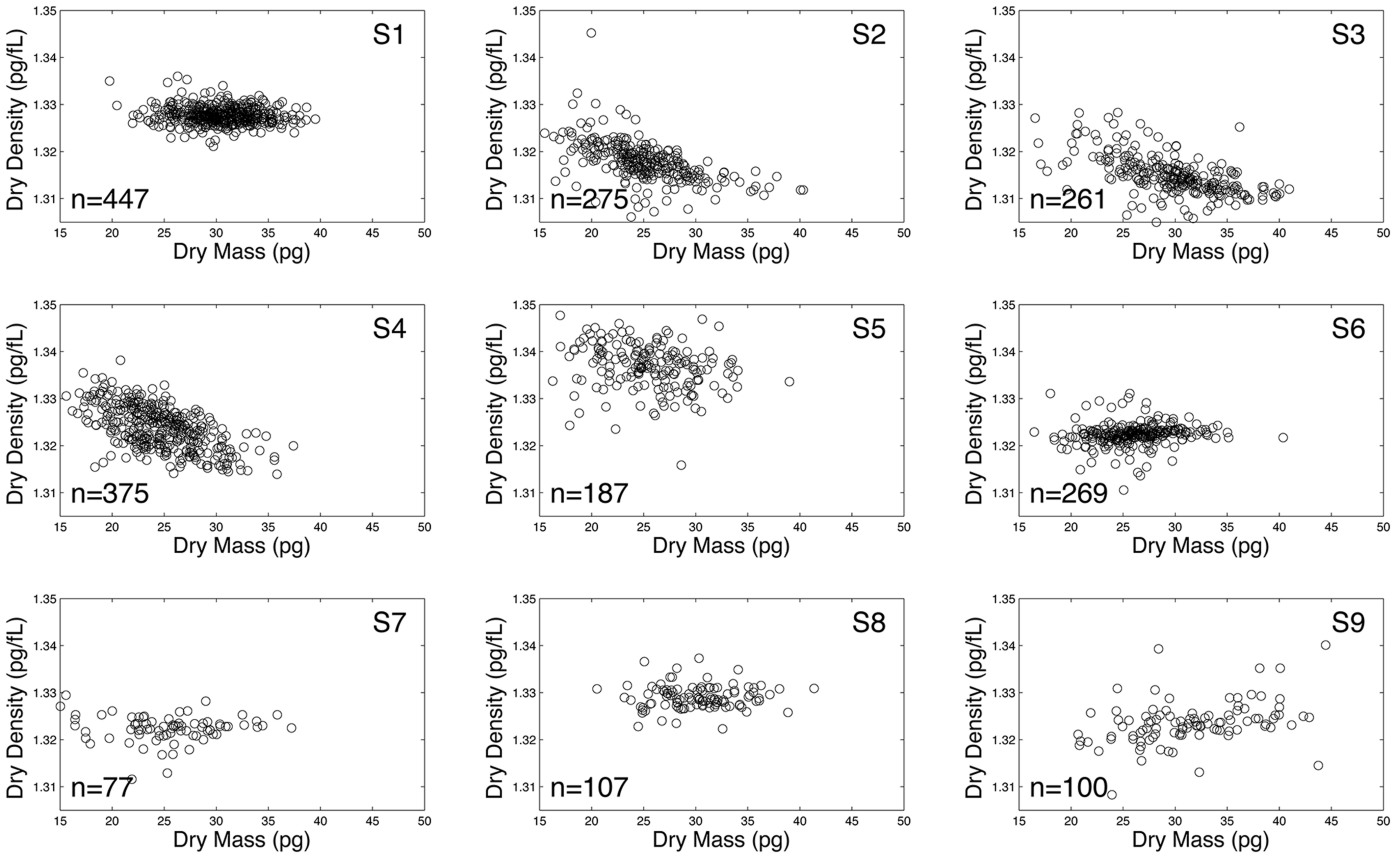
SMR measurements of dry mass and dry density. Each panel (S1-S9) is a population of cells from a single individual.

**Figure 7:**
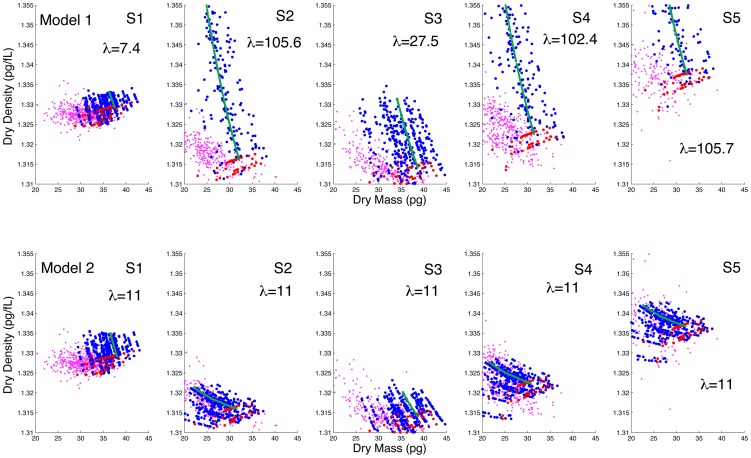
SMR measurements of dry mass and dry density, samples (S1-S5) with the corresponding modeling based on Advia data (first row, model 1, second row model 2, each column is the same SMR sample). For model 1, 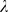 is calculated from the Hb mass model (Eq. (8)), while for model 2 it is fixed to 11. Young cells (red dots) are evolved using either model 1 or 2 to obtain the older cells (blue dots), and compared to the measured SMR data (purple dots). The green line is an example trajectory of a single RBC. Notice that the simulation results (blue dots) are a small random sample from the entire simulated data, matching in size to the SMR sample size.

In Figure 6 we show the raw SMR data: dry mass versus dry density. The young RBC (reticulocyte) data was not collected for all samples and is required for the simulations. In Figure 7 we show those samples with simultaneous measurements of Hb mass and dry density. Here the reported vesiculation rate is calculated per sample according to Eq. (7), adjusted to the Hb mass model (Eq. (8)) for model 1, assuming 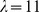 for model 2. The area model (Eq. (9)) can generate an estimate of 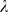, but because we have no individual measurements of cell area, we use 3 single-cell measurements of volume and surface area and match the initial volumes as described above. Theses assumptions probably reduce the variability both within and between samples. The estimates we obtain are in the range 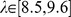. Note that surface area parameters used in Eq. (9) and in Figure 2 are based on Table 4 which is independent of the surface area used in the simulations here, in the context of predicting dry mass-density profiles, that are based on data from [29].

Among the samples in Figure 7, S2 (used in Figure 3), S4, and S5 are more typical of healthy human adults based on the average Hb mass lost. S1 and S3 show a smaller than typical Hb mass loss (Advia measurement). For S3 we see that while the estimated vesiculation rate is only 2.5 times more when estimated using the Hb mass model versus the surface area model, the dry density spans the same range and thus the difference between models 1 and 2 persists. In sample S1 we see that the vesiculation rate is similar when estimated by either the Hb mass or surface area model. In this case the Hb mass lost is 0.33 pg, which is 13.75% of the mean loss in our 21 healthy human adults. Further investigation of this anomaly is beyond the scope of the current study. It is possible that new biophysical measurements like dry-mass and dry-density have diagnostic potential in the context of some forms of anemia which are associated with a reticulocyte population located much closer to the general population (in contrast to Figure 1). It is likely that these pathologic conditions show different RBC maturation patterns.

### Measurement methods review

In this work several data sources have been integrated via our mathematical modeling. We collected the parameter names, methods, and references in Table 7. Here we briefly describe those methods.

The RBC volume and hemoglobin content are measured via the Siemens Advia 2120 automated clinical hematology analyzer [30]. This instrument is essentially a flow cytometer that uses an isovolumetric-sphering reagent prior to the light scattering to render the measurement invariant to cell presentation. Using a pair of small and large angle light scattering intensities and Mie scattering theory, the cell volume and hemoglobin concentration for each cell are calculated.

The surface area is measured using a microfluidic device, either by fixing the cells in a constriction of known geometry [3] or by controlling the flow and utilizing symmetry-based calculations form a 2-dimensional image [29].

The cellular dry mass and dry density are measured using the SMR, a microfabricated mass sensor which implements Archimedes principle in a microfludic device for individual cells. Measuring the buoyant mass, or mass in fluid, of a cell sequentially in two fluids of known density allows inference of the cell's mass, volume, and density. When the two fluids are H_2_O-based and D_2_O-based, the cell exchanges its water content with D_2_O and thus the measurements yield only the dry mass and dry density of the RBC, since only the dry content, and not the aqueous content, contributes to the buoyant mass in either fluids [16].

Vesicle radii were measured either from atomic force microscopy (AFM) images, assuming spherical symmetry, [25] or recently by *μ* NMR [20]. The *μ* NMR method involves labeling micro-vesicles with target-specific (CD235a antibody) magnetic nanoparticles and quantifying their concentration using a miniaturized nuclear magnetic resonance system.

For both RBC lifetime estimation [26] and vesiculation clearance rate [11], [26], [31], [32], the experimental design includes the isolation of RBCs/vesicles from a blood sample, labeling of the isolates, reinfusion of the labeled sample, and measurement of the fraction of labeled cells/vesicles repeatedly over time.

### Ethics statement

The study protocol was approved by the local institutional review board (IRB) at Massachusetts General Hospital, in accordance with the principles expressed in the Declaration of Helsinki. We used blood samples that had been collected solely for non-research purposes (such as medical treatment or diagnosis).
